# Predicting the short and long term effects of food price inflation, armed conflicts, and climate variability on global acute malnutrition in Somalia

**DOI:** 10.1186/s41043-024-00557-9

**Published:** 2024-05-17

**Authors:** Jama Mohamed, Mukhtar Jibril Abdi, Ahmed Ismail Mohamed, Mohamed Aden Muhumed, Barkhad Aden Abdeeq, Abdinasir Ali Abdi, Mohamed Mussa Abdilahi, Dahir Abdi Ali

**Affiliations:** 1 Faculty of Statistics and Data Science, College of Applied and Natural Science, University of Hargeisa, Hargeisa, Somaliland; 2 Center for Ground and Surface Water Management, Hargeisa Water Agency, Hargeisa, Somaliland; 3 Faculty of Nutrition and Food Science, College of Applied and Natural Science, University of Hargeisa, Hargeisa, Somaliland; 4 Department of Planning, Ministry of Planning and Development, Hargeisa, Somaliland; 5 Department of Child Survival, Save the Children International, Hargeisa, Somaliland; 6 College of Business and Public Administration, University of Hargeisa, Hargeisa, Somaliland; 7 College of Medicine and Health Sciences, University of Hargeisa, Hargeisa, Somaliland; 8 Faculty of Economics, SIMAD University, Mogadishu, Somalia

**Keywords:** Global acute malnutrition, Food price inflation, Armed conflicts, Climate variability, Somalia

## Abstract

**Background:**

Malnutrition poses a substantial challenge in Somalia, impacting approximately 1.8 million children. This critical issue is exacerbated by a multifaceted interplay of factors. Consequently, this study seeks to examine the long-term and short-term effects of armed conflicts, food price inflation, and climate variability on global acute malnutrition in Somalia.

**Methods:**

The study utilized secondary data spanning from January 2015 to December 2022, sourced from relevant databases. Two distinct analytical approaches were employed to comprehensively investigate the dynamics of global acute malnutrition in Somalia. Firstly, dynamic autoregressive distributed lag (ARDL) simulations were applied, allowing for a nuanced understanding of the short and long-term effects of armed conflicts, food price inflation, and climate variability on malnutrition. Additionally, the study employed kernel-based regularized least squares, a sophisticated statistical technique, to further enhance the robustness of the findings. The analysis was conducted using STATA version 17.

**Results:**

In the short run, armed conflicts and food price inflation exhibit positive associations with global acute malnutrition, particularly in conflict-prone areas and during inflationary periods. Moreover, climatic variables, specifically temperature and rainfall, demonstrate positive associations. It is important to note that temperature lacks a statistically significant relationship with global acute malnutrition in the short run. In the long run, armed conflicts and food price inflation maintain persistent impacts on global acute malnutrition, as confirmed by the dynamic ARDL simulations model. Furthermore, both temperature and rainfall continue to show positive associations with global acute malnutrition, but it is worth noting that temperature still exhibits a non-significant relationship. The results from kernel-based regularized least squares were consistent, further enhancing the robustness of the findings.

**Conclusions:**

Increased armed conflicts, food price inflation, temperature, and rainfall were associated with increased global acute malnutrition. Strategies such as stabilizing conflict-prone regions, diplomatic interventions, and peace-building initiatives are crucial, along with measures to control food price inflation. Implementing climate adaptation strategies is vital to counter temperature changes and fluctuating rainfall patterns, emphasizing the need for resilience-building. Policymakers and humanitarian organizations can leverage these insights to design targeted interventions, focusing on conflict resolution, food security, and climate resilience to enhance Somalia's overall nutritional well-being.

## Background

Malnutrition remains a pressing global health concern, particularly in regions afflicted by food insecurity, armed conflicts, and climate variability [1–3]. Somalia, one of the countries with the highest child and maternal mortality rates [4], exemplifies a nation grappling with these interrelated challenges. Moreover, it stands as the lowest-ranked country on the food security index, facing the daunting reality of having the highest rate of acute malnutrition in the world [4]. According to recent statistics from the Integrated Food Security Phase Classification (IPC), approximately 1.8 million children under the age of five in Somalia suffered from acute malnutrition in 2023, with the country facing one of the highest burdens of malnutrition globally [5]. Understanding the dynamics of acute malnutrition in such complex environments is crucial for effective policy formulation and intervention strategies.

The relationship between food price inflation and malnutrition has been extensively studied in the literature. High food prices can exacerbate food insecurity, limiting access to nutritious foods, especially among vulnerable populations. Studies like Headey & Ruel [6] and Brinkman et al. [7] highlight the strong link between food price inflation and malnutrition, particularly in children. As food prices rise, especially staples, households struggle to afford enough nutritious food, leading to inadequate calorie intake and increased risk of wasting and stunting. As highlighted by Homeida [8] and Kinyoki et al. [9], fragile settings such as Sudan and Somalia face a critical food security situation compounded by conflict and economic factors. This has significant consequences for vulnerable groups like pregnant women and young children, where increased conflict events contribute to increased wasting and stunting rates [9].

Armed conflicts disrupt food systems, exacerbate poverty, and displace populations, significantly impacting nutritional outcomes, particularly among children. Numerous studies have documented the adverse effects of armed conflicts on malnutrition, emphasizing the role of conflict-induced displacement, destruction of infrastructure, and disruption of livelihoods. For instance, research by Howell et al. [10], Dahab et al. [11], and Kinyoki et al. [9] highlighted how armed conflict disrupts food systems and exacerbates malnutrition. Conflict directly damages agricultural production, destroys infrastructure, and displaces communities, leaving them vulnerable to hunger and undernutrition. This is evident in Somalia, where prolonged conflict has devastated livelihoods and increased reliance on humanitarian aid [9]. The complex and interconnected nature of conflict's impact is emphasized by Dahab et al. [11], who found that while conflict significantly increases malnutrition risk, addressing broader socio-demographic factors remains crucial for effective interventions.

Climate variability, including extreme weather events such as droughts and floods, poses substantial challenges to food security and nutrition, particularly in agrarian societies like Somalia. Studies have highlighted the adverse effects of climate variability on crop production, livestock health, and water availability, consequently influencing food access and nutritional outcomes. For instance, research by Mank et al. [12], Thiede and Gray [13], and Elayouty et al. [14] shed light on the intricate relationship between climate variability and malnutrition. Droughts, floods, and extreme weather events disrupt agricultural production, reduce food availability, and alter dietary patterns, often impacting children the most, as demonstrated by Mank et al. [12]. In Somalia, where water resources are scarce and droughts frequent, climate variability poses an additional threat to food security and nutritional health. Elayouty et al. [14] highlight the need for geographically specific interventions and adaptable risk management strategies to address the varying impacts of climate change on malnutrition across regions.

While existing literature offers valuable insights into the impacts of food price inflation, armed conflicts, and climate variability on global acute malnutrition, there exists a significant gap in understanding within the context of Somalia, where research on this topic is notably limited. Previous global studies have examined the individual effects of food price inflation, climate change, and conflict on malnutrition, yet few have integrated all these variables simultaneously into comprehensive models. This study aims to fill this gap by employing advanced econometric techniques capable of predicting both short-run and long-run effects. Specifically, the study utilizes dynamic AutoRegressive Distributed Lag (ARDL) simulations and kernel-based regularized least squares, offering a more robust framework for understanding the intricate relationships between these factors and acute malnutrition in Somalia. By doing so, this research facilitates evidence-based policymaking and targeted interventions in combating malnutrition in Somalia.

### Motivation and research question

In recent years, Somalia has faced persistent challenges related to global acute malnutrition, exacerbated by a combination of factors such as food price inflation, armed conflicts, and climate variability. Understanding the interplay between these variables is crucial for devising effective interventions to mitigate malnutrition rates and improve the overall well-being of the population. Therefore, the motivation behind this study is to investigate the short and long-term effects of food price inflation, armed conflicts, and climate variability on global acute malnutrition in Somalia.

The primary research question guiding this study is: How do food price inflation, armed conflicts, and climate variability collectively influence global acute malnutrition in Somalia over both short and long-term periods? By examining this question, the study aims to elucidate the specific mechanisms through which these factors interact to affect malnutrition rates. Furthermore, the research explores potential interventions and policy recommendations aimed at mitigating the adverse effects of food insecurity, conflict, and climate change on nutrition outcomes in Somalia. By addressing these critical issues, this study seeks to contribute to the broader discourse on humanitarian aid and development efforts in conflict-affected regions, ultimately striving towards improved health outcomes and resilience among vulnerable populations.

## Methods

### Data source

The data used in this study covered the period from January 2015 to December 2022, providing a total of 96 observations. These data were obtained from diverse and reputable sources to comprehensively examine the intricate dynamics of armed conflicts, food price inflation, and climate variability in shaping global acute malnutrition in Somalia. The confirmed cases of global acute malnutrition (GAM) were obtained from the Food Security and Nutrition Analysis Unit (FSNAU), ensuring a reliable and comprehensive dataset on malnutrition cases in Somalia. Armed conflict data, crucial for understanding conflict-related impacts on malnutrition, was sourced from the Armed Conflict Location and Event Data Project (ACLED). Economic factors influencing malnutrition rates, particularly food price inflation, were extracted from the World Bank dataset, providing essential indicators for a comprehensive analysis. Climate-related variables, including temperature and rainfall, were derived from the Climate Research Unit Time Series (CRU TS) and Climate Hazards Group InfraRed Precipitation with Station (CHIRPS) datasets, respectively. Food price inflation and climatic variables, such as temperature and rainfall, were not aggregated by authors as they are available in monthly time series format from the relevant databases. The reported daily armed conflicts were aggregated by month, and monthly GAM confirmed cases by district. These sources collectively offer a robust foundation for exploring the complex relationships underlying global acute malnutrition in Somalia.

To preprocess the data for analysis, the study utilized natural logarithm form to reduce the variability within the variables. Also, the augmented Dickey–Fuller (ADF), and Phillips–Perron (PP) unit root tests were used to obtain the integration order of series.

### Statistical analysis

#### ARDL model and ARDL bounds testing approach

Autoregressive distributed lag (ARDL) model is a regression framework commonly used in econometrics to analyze the long-run relationships between variables, particularly in time series data. It is a flexible and widely used tool, particularly for investigating issues such as co-integration and causality. The ARDL model offers several advantages compared to conventional approaches for testing co-integration [15]. Firstly, it accommodates scenarios where variables exhibit a combination of stationary (*I*(0)) and non-stationary (*I*(1)) behavior. Secondly, it enables the simultaneous estimation of both short-term and long-term relationships among variables through the ARDL bound testing procedure. Moreover, the ARDL model addresses endogeneity concerns by incorporating lags of both dependent and independent variables within the model structure. On the other hand, the ARDL model is not without limitations. Firstly, the ARDL model assumes linear and symmetric adjustment speeds [16]. Secondly, while the ARDL model attempts to address endogeneity by including lagged values of both dependent and independent variables, it may not fully capture all sources of endogeneity [17]. Lastly, if the variables are highly persistent or exhibit higher orders of integration, the ARDL model may not provide reliable results [17].

The ARDL bounds testing technique, developed by Pesaran et al. [18], is designed to examine long-term relationships among variables with a mixed integration order, specifically *I(*1) or *I*(0), excluding *I*(2). The dependent variable must exhibit an integrated order of *I*(1) in this methodology. Subsequently, the unconstrained error correction model is applied to assess the cointegration of the variables once these criteria are satisfied.

$$\begin{aligned} \ln GAM_{t} & = \delta_{1} + \sum\limits_{i = 1}^{a} {\beta_{1} \Delta \ln GAM_{t - i} } + \sum\limits_{i = 1}^{b} {\beta_{2} \Delta \ln Conflicts_{t - i} } + \sum\limits_{i = 1}^{c} {\beta_{3} \Delta \ln FPI_{t - i} } + \sum\limits_{i = 1}^{d} {\beta_{4} \Delta \ln Temperature_{t - i} } + \sum\limits_{i = 1}^{e} {\beta_{5} \Delta \ln {\text{Rainfall}}_{t - i} } \\ & \quad + \gamma_{1} \ln GAM_{t - 1} + \gamma_{2} \ln Conflicts_{t - 1} + \gamma_{3} \ln FPI_{t - 1} + \gamma_{4} \ln Temperature_{t - 1} + \gamma_{5} \ln {\text{Rainfall}}_{t - 1} + \varepsilon_{t} \\ \end{aligned}$$

Here, Δ represents the difference operator, *δ*_1_ denotes the intercept, and *a*, *b*, *c*, *d*, and *e* represent the selected optimal lags. Short-run coefficients are denoted by *β*_1_, *β*_2_, *β*_3_, *β*_4_, and *β*_5_ while *γ*_1_, *γ*_2_, *γ*_3_, *γ*_4_, and *γ*_5_ illustrate the long-run coefficients. The term *ε*_*t*_ represents the residual.

In Case II, involving restricted intercept and no trend, the alternative and null hypotheses are formulated as follows:

- Null Hypothesis (*H*_0_): *δ*_1_ = *γ*_1_ = *γ*_2_ = *γ*_3_ = *γ*_4_ = *γ*_5_ = 0.
- Alternative Hypothesis (*H*_a_): *δ*_1_ ≠ *γ*_1_ ≠ *γ*_2_ ≠ *γ*_3_ ≠ *γ*_4_ ≠ *γ*_5_ ≠ 0.

Refutation of the null hypothesis, indicating cointegration, occurs if the estimated F-statistic surpasses the critical values' upper bounds determined by Pesaran et al. [18]. Conversely, if the F-statistic does not exceed these critical values, it suggests the absence of a long-term cointegration among the variables.

#### Dynamic ARDL simulations

Complex specifications, encompassing first differences, lagged differences of variables, and diverse lag structures, are commonplace in ARDL modeling. In simpler terms, evaluating the long- and short-run impacts of regressors on the dependent variable proves challenging when utilizing an ARDL model with first differences and multiple lag lengths. To alleviate this challenge, Jordan and Philips [19] introduced dynamic ARDL, integrating a dynamic error correction mechanism. Following the ceteris paribus principle, this approach facilitates the quantification and visual examination of the effects of positive or negative shifts in an independent variable on the dependent variable. Consequently, the dynamic ARDL framework provides a comprehensive assessment of the relationship between dependent and independent variables.

Two prerequisites must be met for the dynamic model's application: the variables must have an integration order of *I*(1) and the the cointegration of the variables is essential [19]. The error correction equation of the dynamic ARDL model is expressed as follows:

$$\begin{aligned} \Delta \ln GAM_{t} & = \theta_{0} + \pi_{0} \ln GAM_{t - 1} + \tau_{1} \Delta \ln Conflicts_{t} + \pi_{1} \ln Conflicts_{t - 1} + \tau_{2} \Delta \ln FPI_{t} + \pi_{2} \ln FPI_{t - 1} \\ & \quad \, + \tau_{3} \Delta \ln temperature_{t} + \pi_{3} \ln temperature_{t - 1} + \tau_{4} \Delta \ln {\text{rainfall}}_{t} + \pi_{4} \ln FPI_{t - 1} + \varepsilon_{t} \\ \end{aligned}$$

with *θ*_0_ representing the constant term, *π*_0_ denoting the error correction term coefficient, *τ*_1_, *τ*_2_, *τ*_3_, and *τ*_4_ illustrating short-term coefficients, and *π*_1_, *π*_2_, *π*_3_, and *π*_4_ representing long-term coefficients. The error term is depicted by ε_*t*_.

#### Kernel-based regularized least squares

Hainmueller and Hazlett [20] introduced the kernel-based regularized least squares approach, offering practical utility in discerning causal-effect relationships by leveraging machine learning algorithms to implement pointwise derivatives. This methodology establishes a modeling framework that adeptly navigates the middle ground between the stringent generalized linear models and the more flexible yet challenging-to-interpret machine learning methods. The kernel-based regularized least squares can be expressed as $\min_{\beta } \left\{ {\sum\limits_{i = 1}^{n} {K_{\lambda } \left( {y_{i} - X_{i} \beta } \right)^{2} + \lambda \sum\limits_{j = 1}^{k} {\beta_{j}^{2} } } } \right\}$. This method enhances the credibility of this study, offering a more comprehensive understanding of the intricate and dynamic relationships underpinning global acute malnutrition in Somalia. Regularization with the kernel-based approach allowed for effective variable selection and model robustness, ensuring reliability in capturing underlying patterns and complexities. The regularization parameter (*λ*) controlled the trade-off between fitting the data and preventing overfitting, emphasizing the model's reliability.

## Results

### Preliminary analysis

**Fig. 1:**
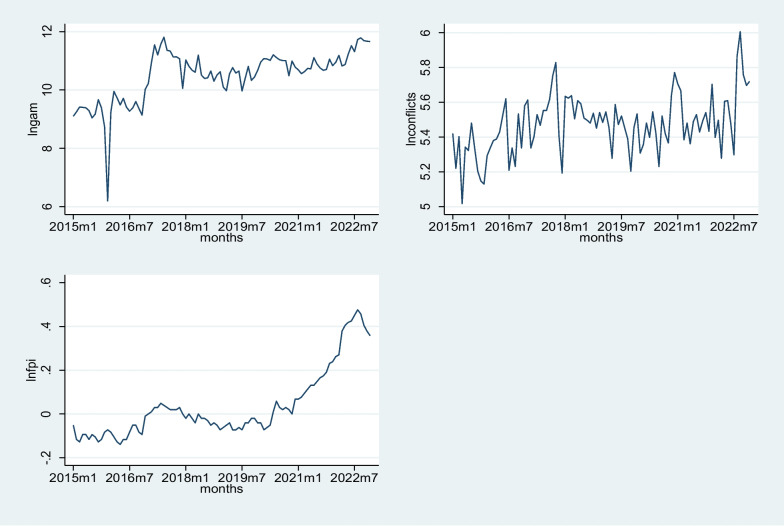
Time series plots of lngam, lnconflicts and lnfpi

To explore and describe the trends in the study variables, time series plots were used. Figure 1 shows the trend patterns in the transformed data using natural log transformation for global acute malnutrition (lngam), armed conflicts (lnconflicts), and food price inflation (lnfpi) over the period from January 2015 to December 2022. The variables are logged to account for potential nonlinear relationships and better capture the underlying patterns. The monthly observations reveal fluctuations and trends in each variable. For instance, in the initial months of 2015, there is a steady increase in the logged global acute malnutrition cases (lngam), while armed conflicts (lnconflicts) show varying patterns with fluctuations. The food price inflation (lnfpi) has negative values that increase slowly, indicating a rising trend. Over subsequent years, the patterns continue to evolve. Noteworthy is the upward trend in global acute malnutrition cases from 2016 to mid-2017, followed by a period of stability and subsequent fluctuations. Armed conflicts exhibit fluctuations with intermittent peaks, and food price inflation shows periods of both rise and fall. The latter part of the dataset, from 2021 to 2022, demonstrates an overall increase in global acute malnutrition, fluctuations in armed conflicts, and food price inflation reaching its peak around mid-2022.

**Fig. 2:**
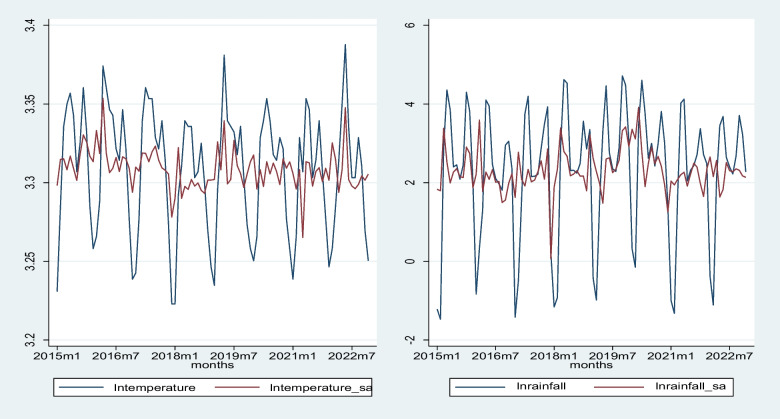
Time series plots of lntemperature and lnrainfall with seasonally adjusted series

Figure 2 illustrates the trend patterns in the transformed variables lntemperature, lnrainfall, lntemperature_sa, and lnrainfall_sa, representing the natural logarithms of temperature, rainfall, seasonally adjusted temperature, and seasonally adjusted rainfall, respectively, over the period from January 2015 to December 2022. Seasonal adjustments to temperature and rainfall account for their seasonal patterns, showing distinct fluctuations over the observed periods. Throughout the period, the lntemperature and lnrainfall exhibit fluctuating patterns, indicating variability in temperature and rainfall levels over the years. The seasonally adjusted temperature and rainfall follow a similar pattern, suggesting that the observed trend is not solely due to seasonal variations.

Table 1 presents summary statistics for the study variables: lngam, lnconflicts, lnfpi, lntemperature_sa, and lnrainfall_sa. For lngam, the mean is 10.48, indicating the average value, and the standard deviation is 0.87, representing the amount of variation around the mean. The minimum and maximum values are 6.19 and 11.81, respectively. The skewness is negative (− 1.46), suggesting a distribution that is skewed to the left, and the kurtosis is 7.40, indicating heavy tails and potential outliers. Similarly, for lnconflicts, the mean is 5.47, with a standard deviation of 0.17. The skewness is positive (0.18), indicating a slight right skewness, and the kurtosis is 3.55. For lnfpi, the mean is 0.04, with a standard deviation of 0.16, skewness of 1.44 (indicating right skewness), and kurtosis of 4.10. For lntemperature_sa, the mean is 3.31, with a standard deviation of 0.01, skewness of 0.42, and kurtosis of 5.55. Lastly, for lnrainfall_sa, the mean is 2.31, with a standard deviation of 0.54, skewness of − 0.06, and kurtosis of 5.81. These statistics provide a comprehensive overview of the central tendency, variability, and distribution shape for each variable, aiding in the interpretation of the dataset. For the variables lngam, lnfpi, lntemperature_sa, and lnrainfall_sa, the *p*-values associated with the chi-squared statistics (111.5, 37.77, 28.87, and 31.55, respectively) are all extremely small (*p* < 0.001), rejecting the null hypothesis (Ho) that the data follows a normal distribution. For lnconflicts, the *p*-value is 0.4271, which is larger but still above the conventional significance level of 0.05. Therefore, while lnconflicts does not show strong evidence of departure from normality, the other variables exhibit significant deviations from a normal distribution based on the Jarque–Bera test.

**Table 1:** Summary statistics

Variable	Mean	Median	Max	Min	SD	Skewness	Kurtosis	J-B stat	*P*-value
lngam	10.4829	10.6776	11.8085	6.1944	0.8743	− 1.4584	7.4023	111.5	< 0.001
lnconflicts	5.4727	5.4806	6.0064	5.017	0.1687	0.1764	3.5486	1.702	0.4271
lnfpi	0.0365	− 0.0202	0.47623	− 0.1393	0.1577	1.4355	4.0954	37.77	< 0.001
lntemperature_sa	3.3087	3.3082	3.3536	3.2652	0.0127	0.4154	5.5547	28.87	< 0.001
lnrainfall_sa	2.3063	2.2668	3.9155	0.0726	0.5398	− 0.0606	5.8057	31.55	< 0.001

The correlation matrix, shown in Table 2, provides insights into the relationships among the study variables. The correlation coefficients, calculated using Spearman correlation due to the non-normal distribution of most variables, reveal several noteworthy patterns. There is a strong positive correlation between lngam and lnfpi (*r* = 0.7719, *p* < 0.01), suggesting that higher levels of food inflation are associated with increased global acute malnutrition. Additionally, lngam and lnconflicts show a moderate positive correlation *(r* = 0.4615, *p* < 0.01), indicating that areas with more conflicts may experience higher malnutrition. Moreover, there is a significant positive correlation between lnfpi and lnconflicts (*r* = 0.4278, *p* < 0.01), implying that higher levels of food price inflation are associated with a greater likelihood of conflicts. The negative correlation between lntemperature_sa and lnfpi (*r* = − 0.2683, *p* < 0.01) suggests that as temperatures decrease, food prices tend to increase. Other correlations, such as lntemperature_sa with lnconflicts and lnrainfall_sa, are relatively weak and may not be statistically significant.

**Table 2:** Correlation matrix

Variables	Lngam	lnconflicts	lnfpi	lntemperature_sa	lnrainfall_sa
Lngam	1.0000	–	–	–	–
lnconflicts	0.4615***	1.0000	–	–	–
lnfpi	0.7719***	0.4278***	1.0000	–	–
lntemperature _sa	− 0.1574	− 0.2476**	− 0.2683***	1.0000	–
lnrainfall_sa	0.1036	− 0.1242	− 0.0717	− 0.1976*	1.0000

*, **, and ***Represent 1%, 5%, and 10% significant levels, respectively

### Unit root tests

Table 3 presents the results from unit root tests for the study variables using the augmented Dickey-Fuller (ADF) and Phillips–Perron (PP) tests. The tests are conducted at both the level and first difference, with consideration given to the presence of intercepts and trends. For the variable lngam, both ADF and PP tests consistently show statistical significance at the 1% level, indicating that global acute malnutrition does not exhibit a unit root. Similarly, lnconflicts, lntemperature_sa, and lnrainfall_sa do not display unit root characteristics, as evidenced by the highly significant test statistics. On the other hand, the variable lnfpi exhibits unit root characteristics, as indicated by non-significant ADF and PP test results, suggesting that food price inflation is not stationary. Overall, these variables show mixed orders of integration, either I(0) or I(1), indicating potential applications for the dynamic ARDL bounds testing approach.

**Table 3:** Results from unit root tests

Variable	ADF		PP	
	*Level*		*Level*	
	*Intercept*	*Intercept and trend*	*Intercept*	*Intercept and trend*
lngam	− 3.153***	− 4.397***	− 2.770*	− 4.234***
lnconflicts	− 5.895***	− 6.803***	− 5.863***	− 6.806***
lnfpi	0.391	− 1.409	0.244	− 1.517
lntemperature_sa	− 7.988***	− 8.504***	− 8.095***	− 8.559***
lnrainfall_sa	− 7.474***	− 7.432***	− 7.495***	− 7.454***
	*First difference*		*First difference*	
	*Intercept*	*Intercept and trend*	*Intercept*	*Intercept and trend*
lngam	− 12.196***	− 12.130***	− 13.467***	− 13.379***
lnconflicts	− 13.956***	− 13.879***	− 16.913***	− 16.803***
lnfpi	− 8.608***	− 8.627***	− 8.601***	− 8.614***
lntemperature_sa	− 15.919***	− 15.831***	− 21.062***	− 20.924***
lnrainfall_sa	− 14.595***	− 14.524***	− 17.893***	− 17.776***

^*^, **, and ***Indicate significance level at 10%, 5%, and 1%, respectively

ADF represents the augmented Dickey–Fuller test and PP represents Phillips–Perron test

### Lag length selection

In Table 4, different lag lengths are assessed using various criteria, and the optimal lag is determined to be lag 3 based on the chosen likelihood ratio (LR) test. While the lag 1 model demonstrates the lowest values for information criteria, such as Akaike information criterion (AIC), Hannan–Quinn information criterion (HQIC), and Schwarz Bayesian information criterion (SBIC), the decision to choose lag 3 is specifically supported by the likelihood ratio test, where the test statistic for lag 3 is 49.495 with 25 degrees of freedom, yielding a *p*-value of 0.002, indicating statistical significance. Therefore, lag 3 is selected due to its superiority in the likelihood ratio test, even though it may not have the absolute lowest values for information criteria.

**Table 4:** Lag length criteria

Lag	LL	LR	df	*p*	FPE	AIC	HQIC	SBIC
0	198.176	–	–	–	1.0e−08	− 4.1995	− 4.1442	− 4.0624
1	414.585	432.82	25	0.000	1.6e−10*	− .3605*	− 8.0286*	− 7.5382*
2	433.035	36.901	25	0.059	1.9e−10	− 8.2182	− 7.6097	− 6.7106
3	457.783	49.495*	25	0.002	1.9e−10	− 8.2127	− 7.3276	− 6.0198
4	469.581	23.597	25	0.543	2.6e−10	− 7.9257	− 6.7640	− 5.0476

*Indicates lag order selected by the criterion

## ARDL model selection and cointegration tests

The ARDL (1,3,0,0,3) model was selected as the optimal model using the AIC metric. The overall model fit in the ARDL model is reflected in the R-squared of 0.7427 and the adjusted R-squared of 0.7078. These values indicate that the model explains approximately 74.27% of the variance in the dependent variable, with the adjusted R-squared accounting for the number of predictors in the model. Then, the ARDL bounds cointegration test was conducted and the results are presented in Table 5 utilizing critical values provided by Kripfganz and Schneider [21]. The calculated F-test statistic is 5.799 is greater than the critical value for I(1) at the 1% significance level, and the associated *p*-value of 0.006 is below conventional thresholds, indicating the rejection of the null hypothesis of no cointegrating relationship at the level (H_0_) in favor of the alternative hypothesis that a level relationship exists (H_1_). Furthermore, the absolute* t*-test statistic of − 5.038 exceeds the I(1) critical value at the 1% level, and the *p*-value for the *t*-test is 0.004, reinforcing the rejection of the null hypothesis of no cointegrating relationship in levels. These results collectively provide strong support for the presence of a cointegrating relationship between the variables.

**Table 5:** ARDL bounds cointegration test

Test statistics	Value	K	*H*_*0*_		*H*_*1*_	
*F*-statistic	5.799	6	No level relationship		Level relationship exists	
*t*-statistic	− 5.038					

Kripfganz and Schneide [21] critical values and approximate *p*-values						
Significance level (%)	F-statistics		t-statistics		I(0)	I(1)
	I(0)	I(1)	I(0)	I(1)	*P*-value for F-statistics	
1	3.970	5.427	− 3.478	− 4.667	0.001	0.006
5	2.946	4.196	− 2.854	− 3.985	*P*-value for *t*-statistics	
10	2.487	3.632	− 2.536	− 3.629	0.000	0.004

## ARDL model estimation

Table 6 presents the results of the ARDL model estimation. The coefficient of − 0.4379 (*p*-value = 0.000) for lngam_t−1_ in the error correction term (ECT) indicates the speed of adjustment, suggesting that about 43.79% of the deviation from the long-run equilibrium is corrected within one period. In the long-run (LR) equation, the coefficients represent the impact of a 1% increase in various factors on the dependent variable (global acute malnutrition). For example, a 1% increase in conflicts at lag 1 (lnconflicts_t−1_) leads to an estimated increase of 4.5895% in global acute malnutrition. Similarly, a 1% increase in lnfpi at lag 1 (lnfpi_t−1_), lntemperature_sa at lag 1 (lntemperature_sa_t−1_), and lnrainfall_sat at lag 1 (lnrainfall_sa_t−1_) is associated with estimated increases of 2.0525, 18.4940, and 0.8175% in the global acute malnutrition, respectively. In the short-run (SR) equation, a 1% increase in ∆lnconflicts leads to a 0.7830% increase in the global acute nutrition, and a 1% increase in ∆lnconflicts at lag 1 (∆lnconflicts_t−1_) is associated with a decrease of 1.2357% in the global acute malnutrition. Similarly, a 1% increase in ∆lnfpi leads to a 0.8988% increase in global acute malnutrition. The short-run coefficients also capture the effects of changes in temperature (∆lntemperature_sa) and rainfall (∆lnrainfall), as well as their lags, on global acute malnutrition. Specifically, a one-percent increase in temperature is associated with an 8.0989% increase in global acute malnutrition, though the result is marginally insignificant at 5% with a *p*-value of 0.066. Similarly, a one-percent increase in rainfall leads to a statistically significant 0.2941% increase in global acute malnutrition.

**Table 6:** ARDL model estimation

	Variable	Coefficient	Std. Error	T	*P*-value	95% CI	
ECT	lngam_t−1_	− 0.4379***	0.0869	− 5.04	0.000	− 0.6109	− 0.2650
LR	lnconflicts_t−1_	4.5895***	1.2112	3.79	0.000	2.1796	6.9995
	lnfpi_t−1_	2.0525**	0.8276	2.48	0.015	0.4059	3.6990
	lntemperature_sa_t−1_	18.4940*	10.5724	1.75	0.084	− 2.5418	39.5298
	lnrainfall_sa_t−1_	0.8175**	0.3483	2.35	0.021	0.1246	1.5104
SR	∆lnconflicts	0.7830**	0.3656	2.14	0.035	0.0556	1.5104
	∆lnconflicts_t−1_	− 1.2357**	0.4932	− 2.51	0.014	− 2.2170	− 0.2545
	∆lnconflicts_t−2_	− 0.5356	0.3655	− 1.47	0.147	− 1.2628	0.1915
	∆lnfpi	0.8988**	0.3922	2.29	0.025	0.1185	1.6791
	∆lntemperature_sa	8.0989*	4.3495	1.86	0.066	− 0.5553	16.7531
	∆lnrainfall_sa	0.2941***	0.1010	2.91	0.005	0.09313	0.4950
	∆lnrainfall_sa_t−1_	− 0.2159*	0.1177	− 1.83	0.070	− 0.4502	0.0184
	∆lnrainfall_sa_t−2_	− 0.2127**	0.0963	− 2.21	0.030	− 0.4043	− 0.0210
	Constant	− 34.0304**	15.7759	− 2.16	0.034	− 65.4196	− 2.6413

*, **, and ***Indicate significance level at 10%, 5%, and 1%, respectively

### Diagnostic checking

**Fig. 3:**
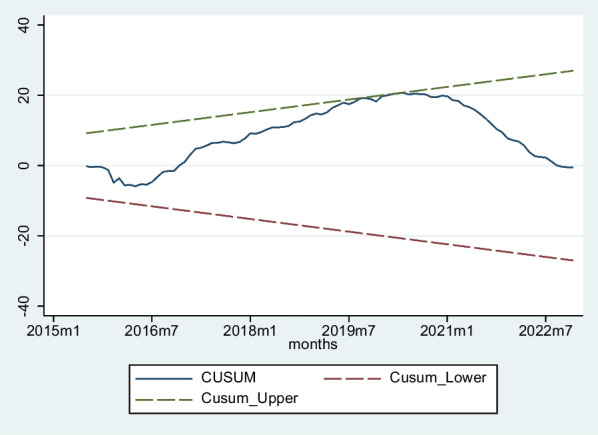
Plot of cumulative sum of recursive residuals

**Fig. 4:**
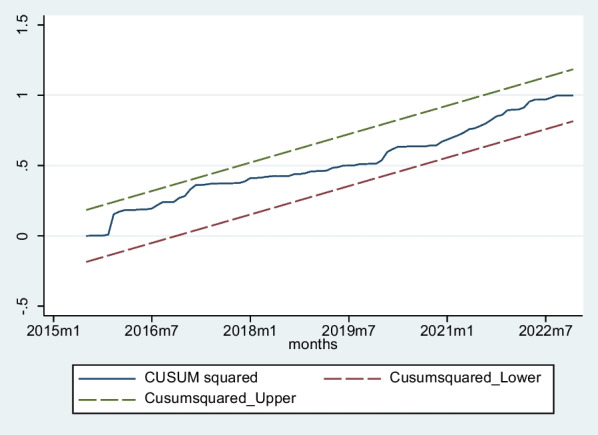
Plot of cumulative sum of squares of recursive residuals

Table 7 presents the results of diagnostic statistical tests for the ARDL model, assessing various aspects of model validity. The Breusch Godfrey LM test with a chi-square statistic of 0.896 and 1 degree of freedom yields a* p*-value of 0.8315, indicating that there is no evidence of serial correlation in the model residuals. This suggests that the residuals do not exhibit a systematic pattern of correlation, supporting the model's appropriateness for capturing the relationships among the variables. White's test for heteroskedasticity, with a chi-square statistic of 88.19 and 77 degrees of freedom, yields a *p*-value of 0.1803, indicating the absence of heteroskedasticity in the residuals. The Cameron–Trivedi tests for skewness and kurtosis produce chi-square statistics of 11.37 (11 degrees of freedom) and 1.19 (1 degree of freedom) with *p*-values of 0.4131 and 0.2761, respectively. These results suggest that the model residuals exhibit no significant skewness or kurtosis, meaning that residuals do not deviate from normality. Additionally, as evidenced in Fig. 3 and Fig. 4, the cusum and cusum squares of recursive residuals are within acceptable limits, further affirming the stability and adequacy of the ARDL model.

**Table 7:** Diagnostic statistical tests

Diagnostic test	Chi-square statistic	df	*P*-value	Conclusion
Breusch Godfrey LM test	0.896	1	0.8315	No serial correlation
White’s test	88.19	77	0.1803	No heteroskedasticity
Cameron-Trivedi test of skewness	11.37	11	0.4131	No skewness
Cameron-Trivedi test of kurtosis	1.19	1	0.2761	No kurtosis

### Dynamic ARDL simulations model results

The dynamic ARDL simulation results in Table 8 reveal significant relationships between the global acute malnutrition and various factors. In the lagged levels equation, a 1% increase in the global acute malnutrition at lag 1 (lngam_t−1_) is associated with a 0.4328% decrease in global acute malnutrition in the current period, indicating a self-correcting mechanism. Furthermore, conflicts at lag 1 (lnconflicts_t−1_) exhibit a positive impact on global acute malnutrition, with a 1% increase resulting in a 1.9082% increase in global acute malnutrition. Similarly, the food price inflation at lag 1 (lnfpi_t−1_) shows a positive association, contributing to a 0.8724% increase in global acute malnutrition for a 1% increase. Temperature at lag 1 (lntemperature_sa_t−1_) do not show statistically significant effect on global acute malnutrition at 5% level of significance while rainfall at lag 1 (lnrainfall_sa_t−1_) exhibits statistically significant effect.

**Table 8:** Dynamic simulated ARDL model estimation

Variable	Coefficient	Std. Error	t	*P*-value	95% CI	
lngam_t−1_	− 0.4328***	0.0880	− 4.92	0.000	− 0.6080	− 0.2578
lnconflicts_t−1_	1.9082***	0.6755	2.82	0.006	0.5635	3.2528
lnfpi_t−1_	0.8724**	0.4122	2.12	0.037	0.0519	1.6929
lntemperature_sa_t−1_	5.3247	5.8408	0.91	0.365	− 6.3011	16.9505
lnrainfall_sa_t−1_	0.3541**	0.1632	2.17	0.033	0.0292	0.6790
∆lnconflicts	0.7543**	0.3768	2.00	0.049	0.0044	1.5043
∆lnconflicts_t−1_	− 1.2001**	0.5068	− 2.37	0.020	− 2.2089	− 0.1913
∆lnconflicts_t−2_	− 0.4491	0.3854	− 1.17	0.247	− 1.2162	0.3180
∆lnfpi	1.1530	1.9861	0.558	0.563	− 2.8003	5.1063
∆lntemperature_sa	8.5955*	4.5026	1.91	0.060	− 0.3667	17.5578
∆lnrainfall_sa	0.3010***	0.1023	2.94	0.004	0.0974	0.5046
∆lnrainfall_sa_t−1_	− 0.2213*	0.1190	− 1.86	0.067	− 0.4581	0.0156
∆lnrainfall_sa_t−2_	− 0.2169**	0.0973	− 2.23	0.029	− 0.4106	− 0.0232
Constant	− 24.3393	21.3393	− 1.16	0.250	− 66.1752	17.4966

*, **, and ***Indicate significance level at 10%, 5%, and 1%, respectively

In the short-run differences equation, changes in conflicts (∆lnconflicts), especially at lag 1 (∆lnconflicts_t−1_), play a significant role. A 1% increase in ∆lnconflicts leads to a 0.7543% increase in global acute malnutrition, while a 1% increase in ∆lnconflicts_t−1_ corresponds to a decrease of 1.2001% in global acute malnutrition. Food price inflation (∆lnfpi) and changes in temperature (∆lntemperature_sa) do not demonstrate statistically significant impacts on global acute malnutrition. However, changes in rainfall at lag 0 (∆lnrainfall_sa) have a significant positive effect, contributing to a 0.3010% increase in global acute malnutrition for a 1% change. Lagged changes in rainfall at lags 1 and 2 (∆lnrainfall_sa_t−1_ and ∆lnrainfall_sa_t−2_) also show significance, with a negative impact on global acute malnutrition.

**Fig. 5:**
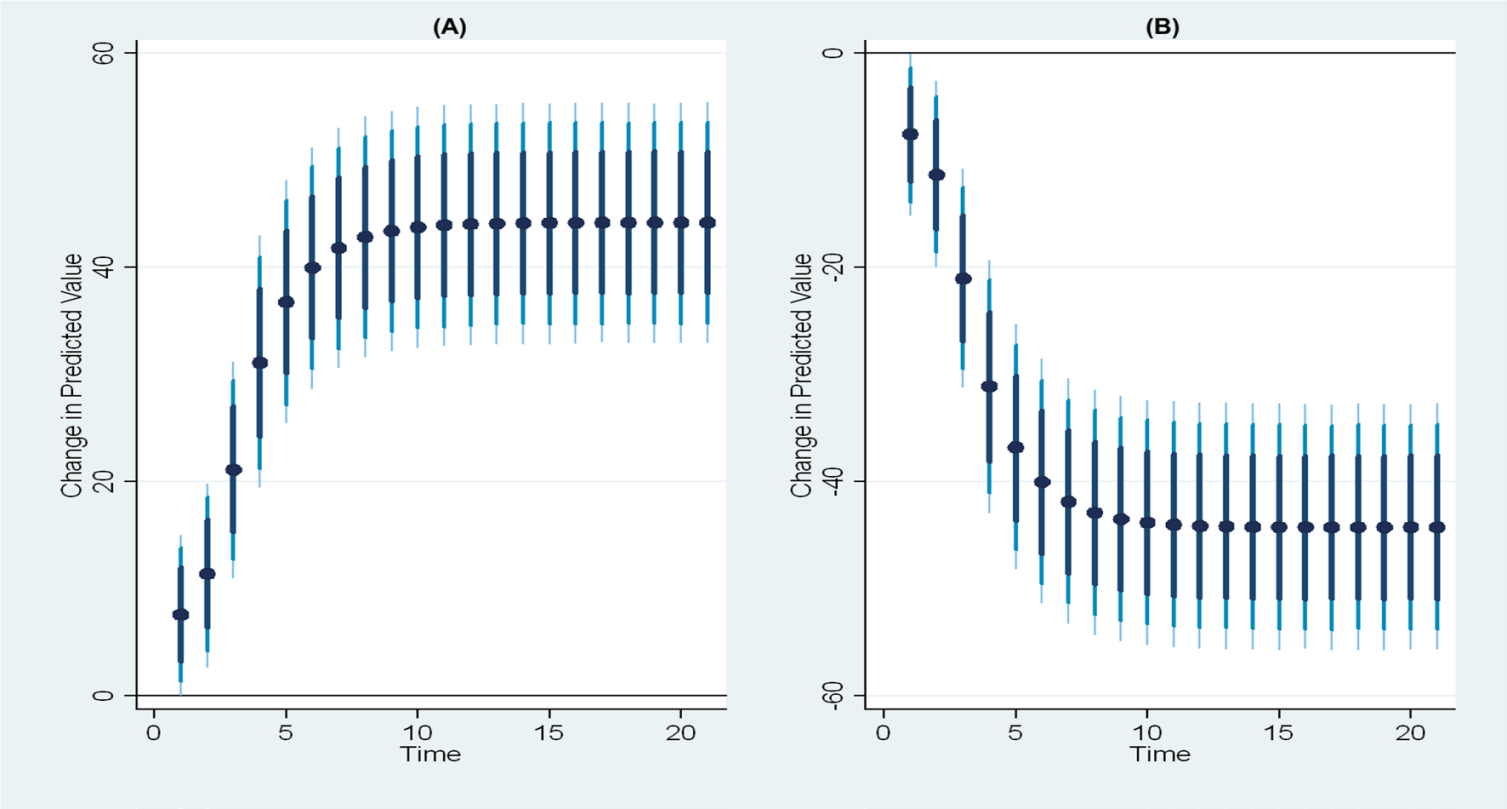
Impulse response plot depicting the scale effect (armed conflicts) and its influence on global acute malnutrition. Panels **a** and **b** illustrate the impact of a 10% increase and decrease in the scale effect on global acute malnutrition, respectively, with dots indicating average prediction values. The dark blue to light blue lines represent 75%, 90%, and 95% confidence intervals

**Fig. 6:**
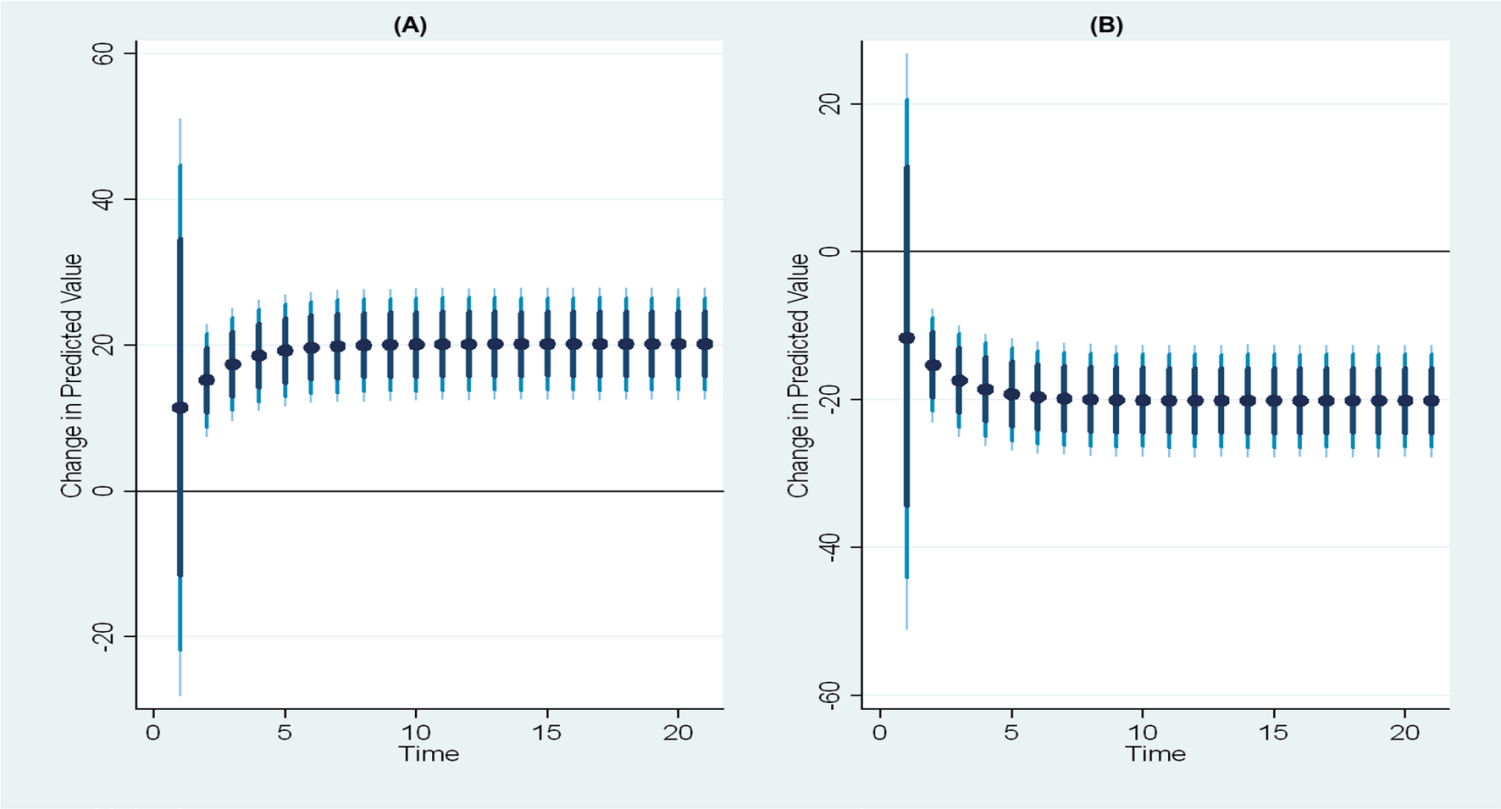
Impulse response plot depicting the scale effect (food price inflation) and its influence on global acute malnutrition. Panels **a** and **b** illustrate the impact of a 10% increase and decrease in the scale effect on global acute malnutrition, respectively, with dots indicating average prediction values. The dark blue to light blue lines represent 75%, 90%, and 95% confidence intervals

The dynamic ARDL model possesses a crucial capability to simulate and predict counterfactual changes in the regressor, capturing the impact of shocks on global acute malnutrition. Each figure in the analysis represents a 10% increment or reduction in the regressor, holding all other factors constant. Dark blue dots signify the expected value, while the dark blue to light blue lines delineate confidence intervals at 75%, 90%, and 95%. The first trend line in the graph highlights short-term effects, while the horizontal line portrays long-term effects over time. Figure 5 reveals that a 10% alteration in armed conflicts has a noteworthy short-term impact on global acute malnutrition. Nevertheless, over time, a 10% increase in armed conflicts leads to a positive escalation in global acute malnutrition and a 10% decrease results in a reduction. This impact is more pronounced in the long term due to the larger marginal rate of rise from the baseline. Figure 6 indicates that a 10% surge or decline in food price inflation lacks a significant short-term effect on global acute malnutrition, while its influence becomes apparent in the long term. However, changes in food price inflation significantly affect global acute malnutrition in the long run, as evidenced by the dotted line's tendency to deviate from the baseline over time.

**Fig. 7:**
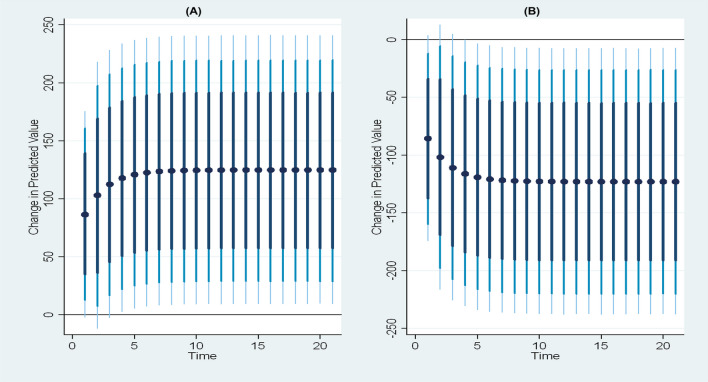
Impulse response plot depicting the scale effect (seasonally adjusted temperature) and its influence on global acute malnutrition. Panels **a** and **b** illustrate the impact of a 10% increase and decrease in the scale effect on global acute malnutrition, respectively, with dots indicating average prediction values. The dark blue to light blue lines represent 75%, 90%, and 95% confidence intervals

**Fig. 8:**
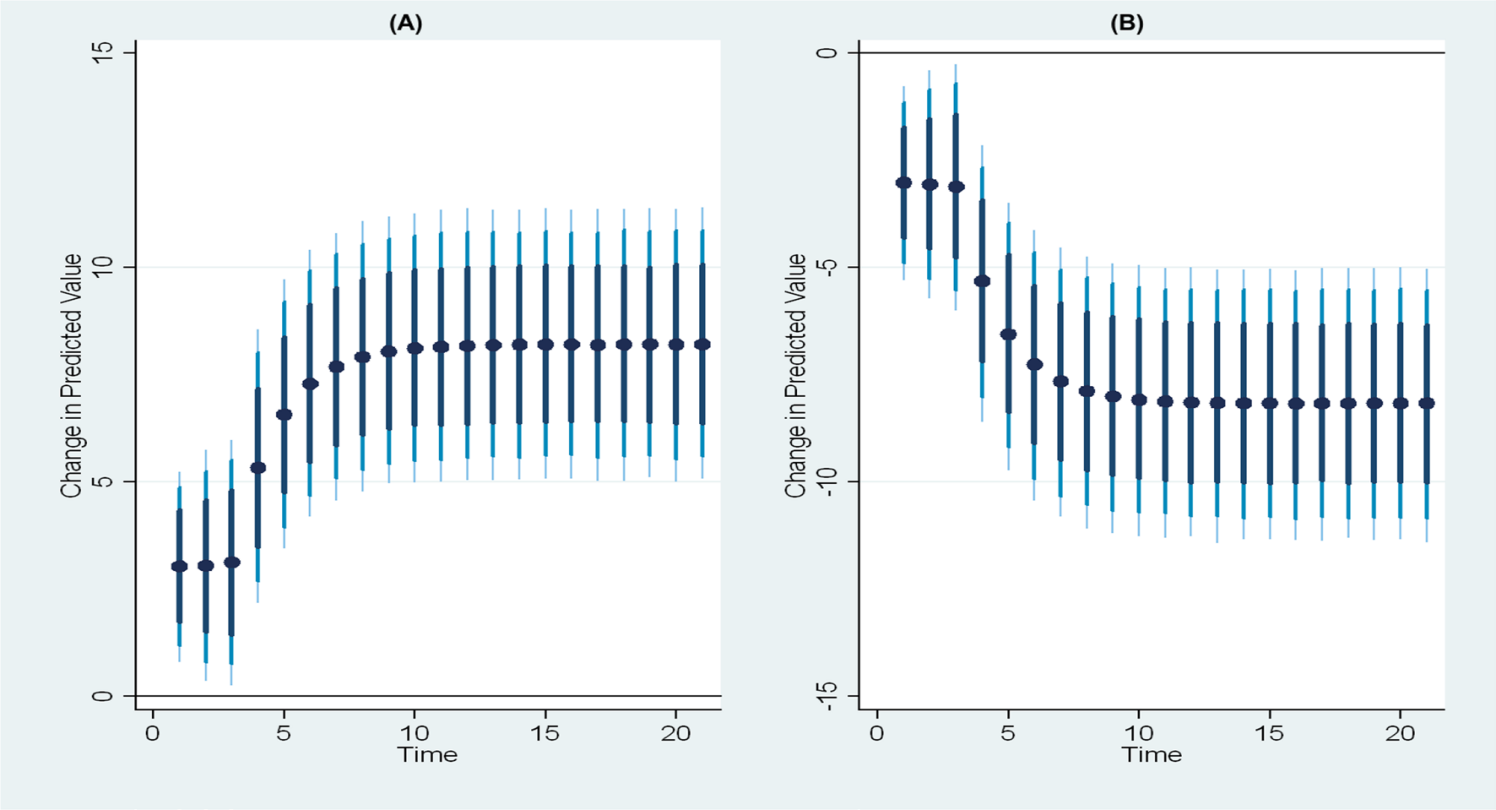
Impulse response plot depicting the scale effect (seasonally adjusted rainfall) and its influence on global acute malnutrition. Panels **a** and **b** illustrate the impact of a 10% increase and decrease in the scale effect on global acute malnutrition, respectively, with dots indicating average prediction values. The dark blue to light blue lines represent 75%, 90%, and 95% confidence intervals

Moving on to Fig. 7, a 10% increase or decrease in seasonally adjusted temperature has a substantial short- and long-term impact on global acute malnutrition. Despite both scenarios having a marginal effect on malnutrition, mitigating malnutrition can benefit more from a 10% decrease in temperature, while a temperature increase exacerbates the malnutrition situation in Somalia. Additionally, Fig. 8 illustrates the 10% positive and negative changes in seasonally adjusted rainfall and their impacts on global acute nutrition in Somalia. It is evident from the figure that a 10% positive change in seasonally adjusted rainfall increases global acute nutrition in both the short and long run, with the most significant effect observed in the long run. Conversely, the reduction in seasonally adjusted rainfall can mitigate malnutrition by decreasing displacement due to floods.

### Kernel-based regularized least squares

Table 9 presents the pointwise derivatives obtained through kernel-based regularized least squares, providing insights into the sensitivity of global acute malnutrition to changes in the explanatory variables. The average pointwise derivative for each variable is accompanied by its standard error, t-value, and *p*-value. For lnconflicts, the average pointwise derivative is 1.3078 with a standard error of 0.3132, indicating a statistically significant positive relationship (*t* = 4.175, *p* < 0.001). The variability in lnconflicts contributes to variations in global acute malnutrition. Similarly, for lnfpi, the average pointwise derivative is 4.1314 with a standard error of 0.4608, suggesting a significant positive relationship (*t* = 8.965, *p* < 0.001). Changes in lnfpi have a substantial impact on the global acute malnutrition. In the case of lntemperature_sa, the average pointwise derivative is 5.0174 with a standard error of 4.3902, indicating a positive relationship, although it is not statistically significant at the conventional significance level (*t* = 1.143, *p* = 0.256). The wide standard error suggests some uncertainty in the estimated relationship. For lnrainfall_sa, the average pointwise derivative is 0.3305 with a standard error of 0.1046, suggesting a statistically significant positive relationship (*t* = 3.161, *p* = 0.002). Changes in lnrainfall_sa have a notable impact on the global acute malnutrition.

**Table 9:** Pointwise derivatives using kernel-based regularized least squares

Variable	Average	SE	*t*	*P*-value	P25	P50	P75
lnconflicts	1.3078	0.3132	4.175	0.000	0.0623	1.1499	2.4748
lnfpi	4.1314	0.4608	8.965	0.000	2.0163	4.3750	6.4836
lntemperature_sa	5.0174	4.3902	1.143	0.256	− 11.5978	4.0170	19.8267
lnrainfall_sa	0.3305	0.1046	3.161	0.002	0.0123	0.3522	0.6612

Diagnostics							
Lambda	0.3045	Sigma	4	R^2^	0.748	Obs	96
Tolerance	0.0960	Eff. Df	28.77	Looloss	36.69		

The diagnostics section in Table 9 provides additional information. The lambda value, a regularization parameter, is 0.3045, indicating the degree of regularization applied in the estimation process. Sigma, representing the bandwidth, is 4. The model's goodness of fit is reflected in an R-squared of 0.748, indicating that approximately 74.8% of the variability in the global acute malnutrition is explained by the explanatory variables. The effective degrees of freedom are 28.77, reflecting the model's complexity. The leave-one-out loss (Looloss) is 36.69, providing a measure of model fit.

**Fig. 9:**
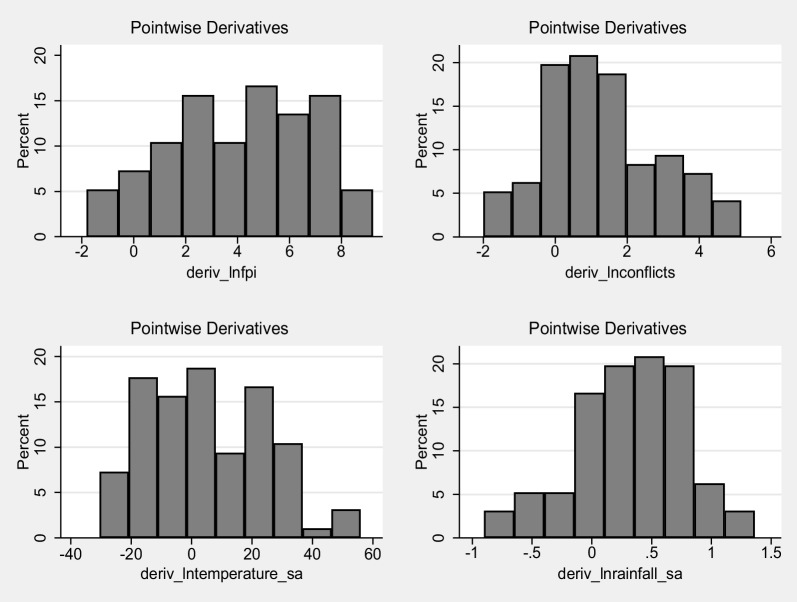
Distribution of pointwise marginal effects of food price inflation, armed conflicts, temperature, and rainfall on global acute malnutrition

Another method for promptly assessing the heterogeneity of effects is the presentation of a histogram of the pointwise marginal effects, as depicted in Fig. 9. The confirmation of substantial effect heterogeneity is obtained from the histogram, indicating that the average marginal effects provide only partial information regarding the diverse effects of armed conflicts, food price inflation, temperature, and rainfall on global acute malnutrition.

**Fig. 10:**
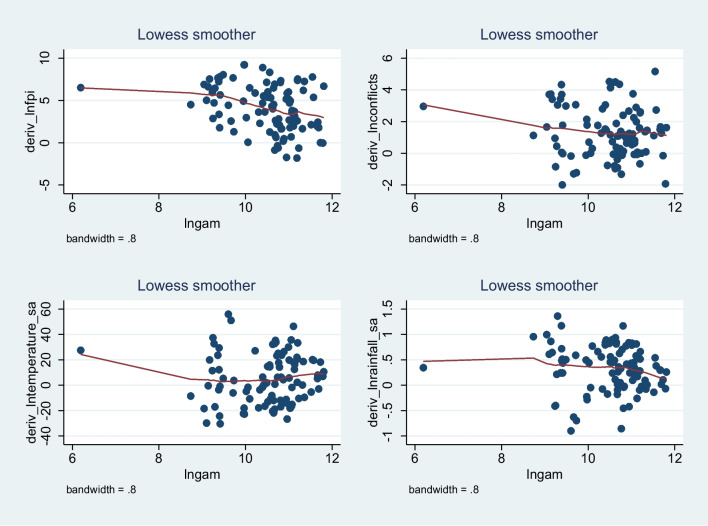
Representation of Pointwise marginal effects of food price inflation, armed conflicts, temperature, and rainfall on global acute malnutrition

Furthermore, an examination of how and why marginal effects vary for armed conflicts, food price inflation, temperature, and rainfall were conducted. This was achieved by plotting the marginal effects against the levels of armed conflicts, food price inflation, temperature, and rainfall. The results, depicted in Fig. 10, showcase how the marginal effect estimates from kernel-based regularized least squares effectively trace the derivative of the nonlinear conditional relationship. The observation reveals that the marginal effect is generally negative at low levels of each variable and becomes positive at high levels of each variable.

## Discussion

The main aim of this study is to conduct a comprehensive examination of the intricate dynamics involving armed conflicts, food price inflation, and climate variability, and their combined impact on global acute malnutrition in Somalia. Employing sophisticated analytical models such as dynamic ARDL simulations and kernel-based regularized least squares, the research delves into the nuanced interactions within these variables to unravel their intricate relationships. In the short run, the discerned positive associations between armed conflicts, food price inflation, and malnutrition not only highlight the vulnerability of conflict-prone regions but also underscore the heightened risk during periods of economic inflation. This aligns seamlessly with prior research that emphasizes the amplified threat of malnutrition in areas affected by armed conflicts and economic instability, contributing to the broader discourse on the far-reaching consequences of socio-economic disruptions [6, 7, 9–11, 22].

Furthermore, in the short run, the positive associations between climatic variables (temperature and rainfall) and global acute malnutrition imply a multifaceted relationship influenced by environmental factors. Elevated temperatures and increased rainfall may exacerbate malnutrition levels, potentially due to their impact on agricultural productivity and food security. This observation aligns with studies emphasizing the vulnerability of susceptible populations to climate-induced food insecurity and malnutrition [12, 13, 23, 24]. Some studies suggest that heightened rainfall, particularly resulting from floods, might displace populations, thereby increasing vulnerability to malnutrition by disrupting access to food, water, and healthcare. Additionally, while elevated temperatures can influence crop yields, extreme heat events could negatively impact agricultural productivity, contributing to food shortages and malnutrition [25]. It is essential to note that certain studies underscore the increased likelihood of malnutrition with rising temperatures and decreasing rainfall [26–28], emphasizing the role of geographical context and infrastructural differences between countries.

The examination of the short-term dynamics reveals a notable absence of a significant relationship between temperature and malnutrition, prompting a deeper exploration of the intricate interactions between climate variables and nutritional outcomes. This intriguing result challenges conventional assumptions and underscores the necessity for more nuanced, context-specific investigations. Notably, this finding aligns with recent studies advocating for a localized understanding of climate impacts on nutrition, as emphasized by Fanzo et al. [29].

In contrast, the long-term analysis using the ARDL model sheds light on the enduring consequences of armed conflicts and food price inflation on global acute malnutrition. This persistence of impacts underscores the critical need for sustained efforts to address conflict and enhance economic stability, as these factors play pivotal roles in mitigating long-term malnutrition challenges. These findings resonate with the arguments put forth by Homeida [8] and Fadare et al. [30], emphasizing the multifaceted nature of interventions required for sustained nutritional well-being.

Furthermore, the enduring positive associations between temperature, rainfall, and malnutrition in the long run underscore the lasting impact of climate variability on nutritional outcomes. This observation is consistent with an expanding body of literature recognizing the role of climate change in shaping long-term nutritional patterns. Studies by van der Merwe et al. [28], Muttarak and Dimitrova [31], and Elayouty et al. [14] have contributed to this growing understanding. However, the non-significant positive relationship between temperature and malnutrition in the long run poses a significant avenue for further investigation, challenging prevailing assumptions about the linear nature of climate-malnutrition associations.

The robustness of these findings is further supported by the consistent results obtained through kernel-based regularized least squares. This analytical approach enhances the credibility of the study, providing a more comprehensive understanding of the complex relationships underpinning global acute malnutrition in Somalia. The application of kernel-based regularized least squares not only ensures the stability and replicability of our results but also contributes to the reliability of the observed patterns and dynamics within the context of our study. This cutting-edge technique goes beyond traditional analyses and positions our research at the forefront of methodological advancements in nutritional studies, ensuring a more thorough and nuanced exploration of the complex relationships within our study.

Despite the robustness of the findings, it is crucial to acknowledge certain limitations in the current study. Firstly, the study relies on secondary data, which introduces inherent limitations, such as the lack of control over the data collection processes. While efforts were made to ensure the reliability and validity of the data sources, the potential for errors or biases inherent in secondary data cannot be completely eliminated. Additionally, the analysis spans a considerable timeframe, from January 2015 to December 2022, and variations in data quality and availability over this period may potentially influence the results. It is important to note that the data may underreport global acute malnutrition in Somalia due to potential gaps or inconsistencies in reporting mechanisms. Furthermore, the complex nature of conflict dynamics and the multifaceted impact of climate variability pose significant challenges in capturing the full spectrum of influencing factors accurately. While the study attempted to account for these complexities through sophisticated analytical techniques, there may still be unobservable factors or interactions that were not fully accounted for in the analysis. Moreover, the study focused primarily on quantitative data, limiting the exploration of qualitative factors that may also play a significant role in shaping global acute malnutrition outcomes. Additionally, we did not account for regional differences, which could have an impact on the results and their generalizability to different parts of Somalia. To address these limitations and further enrich the understanding of the subject, future research endeavors should consider regional differences, incorporating more nuanced data sources, extending the time span under investigation to capture long-term trends and dynamics, and integrating qualitative insights through approaches such as interviews or focus group discussions with key stakeholders. This holistic approach will contribute to a more comprehensive and nuanced exploration of the determinants and dynamics of global acute malnutrition in the context of Somalia, ultimately informing more effective policy and intervention strategies.

## Conclusions and recommendations

The study employs dynamic ARDL simulations and kernel-based regularized least squares to analyze the long and short-term effects of armed conflicts, flood price inflation, and climate variability on global acute malnutrition in Somalia. In the short run, armed conflicts and food price inflation exhibit positive associations with global acute malnutrition, indicating higher malnutrition rates in conflict-prone areas and during periods of inflation. Moreover, climatic variables (temperature and rainfall) show positive associations with global acute malnutrition, suggesting that elevated temperatures and rainfall may exacerbate malnutrition levels. However, temperature does not exhibit a statistically significant relationship with global acute malnutrition in the short run. In the long run, armed conflicts and food price inflation maintain persistent impacts on global acute malnutrition, as evidenced by the dynamic ARDL simulations model. Temperature and rainfall continue to demonstrate positive associations with global acute malnutrition, emphasizing their potential role in long-term nutritional deteriorations. Temperature, while not significant in the short run, still does not not exhibit a statistically significant positive relationship with global acute malnutrition in the long run. The diagnostic checks affirm the model's validity, supporting its appropriateness for capturing complex relationships among variables. These results are consistent with those from kernel-based regularized least squares further enhancing the robustness of these findings.

Recommendations arising from these findings emphasize the importance of addressing armed conflicts, food price inflation, and climate variability to mitigate global acute malnutrition in Somalia. Efforts to stabilize regions prone to conflicts can significantly reduce malnutrition rates, suggesting the need for diplomatic interventions and peace-building initiatives. Additionally, measures to control and manage food price inflation are crucial, as higher inflation levels are associated with increased malnutrition. Climate adaptation strategies should be implemented to mitigate the adverse effects of temperature changes and fluctuating rainfall patterns, emphasizing the importance of building resilience against climate-related shocks. Policymakers and humanitarian organizations can apply these findings to design specific interventions that prioritize conflict resolution, food security, and climate resilience, ultimately contributing to an improvement in Somalia's comprehensive nutritional health. Furthermore, it is essential to enhance data collection and monitoring systems to track changes in armed conflicts, food prices, and climatic conditions, enabling timely interventions and targeted resource allocation to the most vulnerable populations. Public health campaigns and community-based interventions should also be strengthened to promote nutrition education, breastfeeding practices, and access to fortified foods, especially in conflict-affected and food-insecure areas. By adopting a multi-sectoral approach that addresses the underlying drivers of malnutrition, stakeholders can work towards sustainable solutions that improve the overall health and well-being of Somalia's population.

## Data Availability

The data utilized in this study can be accessed from various reputable sources. The confirmed cases of Global Acute Malnutrition (GAM) were acquired from the Food Security and Nutrition Analysis Unit (FSNAU) [http://www.fsnau.org/]. Armed conflict data, essential for comprehending conflict-related impacts on malnutrition, was obtained from the Armed Conflict Location & Event Data Project (ACLED) [https://acleddata.com/]. Economic factors influencing malnutrition rates, particularly food price inflation, were extracted from the World Bank dataset [https://data.worldbank.org/]. Climate-related variables, including temperature and rainfall, were derived from the Climate Research Unit Time Series (CRU TS) [https://crudata.uea.ac.uk/cru/data/hrg/] and Climate Hazards Group InfraRed Precipitation with Station (CHIRPS) datasets [https://www.chc.ucsb.edu/data].
